# *Dusp3* and *Psme3* Are Associated with Murine Susceptibility to *Staphylococcus aureus* Infection and Human Sepsis

**DOI:** 10.1371/journal.ppat.1004149

**Published:** 2014-06-05

**Authors:** Qin Yan, Batu K. Sharma-Kuinkel, Hitesh Deshmukh, Ephraim L. Tsalik, Derek D. Cyr, Joseph Lucas, Christopher W. Woods, William K. Scott, Gregory D. Sempowski, Joshua Thaden, Thomas H. Rude, Sun Hee Ahn, Vance G. Fowler

**Affiliations:** 1 Division of Infectious Diseases & International Health, Department of Medicine, Duke University School of Medicine, Durham, North Carolina, United States of America; 2 Division of Neonatology, Department of Pediatrics, Perelman School of Medicine, University of Pennsylvania, Philadelphia, Pennsylvania, United States of America; 3 Emergency Medicine Service, Durham Veteran's Affairs Medical Center, Durham, North Carolina, United States of America; 4 Duke Institute for Genome Sciences & Policy, Duke University, Durham, North Carolina, United States of America; 5 Quintiles Innovations, Morrisville, North Carolina, United States of America; 6 Section on Infectious Diseases, Durham Veteran's Affairs Medical Center, Durham, North Carolina, United States of America; 7 Hussman Institute for Human Genomics, University of Miami Miller School of Medicine, Miami, Florida, United States of America; 8 Dr. John T. Macdonald Foundation Department of Human Genetics, University of Miami Miller School of Medicine, Miami, Florida, United States of America; 9 Duke Human Vaccine Institute, Durham, North Carolina, United States of America; 10 Duke Clinical Research Institute, Durham, North Carolina, United States of America; 11 Department of Biochemistry School of Dentistry, Chonnam National University, Bukgu, Gwangju, Korea; Vanderbilt University, United States of America

## Abstract

Using A/J mice, which are susceptible to *Staphylococcus aureus*, we sought to identify genetic determinants of susceptibility to *S. aureus*, and evaluate their function with regard to *S. aureus* infection. One QTL region on chromosome 11 containing 422 genes was found to be significantly associated with susceptibility to *S. aureus* infection. Of these 422 genes, whole genome transcription profiling identified five genes (*Dcaf7*, *Dusp3*, *Fam134c*, *Psme3*, and *Slc4a1*) that were significantly differentially expressed in a) *S. aureus* –infected susceptible (A/J) vs. resistant (C57BL/6J) mice and b) humans with *S. aureus* blood stream infection vs. healthy subjects. Three of these genes (*Dcaf7, Dusp3, and Psme3*) were down-regulated in susceptible vs. resistant mice at both pre- and post-infection time points by qPCR. siRNA-mediated knockdown of *Dusp3* and *Psme3* induced significant increases of cytokine production in *S. aureus*-challenged RAW264.7 macrophages and bone marrow derived macrophages (BMDMs) through enhancing NF-κB signaling activity. Similar increases in cytokine production and NF-κB activity were also seen in BMDMs from CSS11 (C57BL/6J background with chromosome 11 from A/J), but not C57BL/6J. These findings suggest that *Dusp3* and *Psme3* contribute to *S. aureus* infection susceptibility in A/J mice and play a role in human *S. aureus* infection.

## Author Summary

*Staphylococcus aureus* causes life-threatening infections in humans. Host genetic determinants influence the outcome of *S. aureus* infection, yet are poorly understood. Susceptible A/J and resistant C57BL/6J mice provide a unique platform to study the genetic difference responsible for variable host response to *S. aureus* infection. We showed that chromosome 11 in A/J was responsible for susceptibility to *S. aureus*. We further identified a QTL locus on Chromosome 11 significantly associated with *S. aureus* susceptibility. Five genes in the QTL (*Dcaf7*, *Dusp3*, *Fam134c*, *Psme3*, and *Slc4a1*) were significantly differently expressed in a) susceptible vs. resistant mice, and b) humans with *S. aureus* blood stream infection vs. healthy human subjects. Three genes (*Dusp3*, *Psme3*, and *Dcaf7*) were down-regulated in susceptible A/J mice. siRNA-mediated knockdown of *Dusp3* and *Psme3* in bone marrow derived macrophage (BMDMs) significantly enhanced cytokine responses through NF-κB activity upon *S. aureus* challenge in a pattern that was also present in *S. aureus*-challenged BMDMs from susceptible CSS11 (chr. 11 from A/J but otherwise C57BL/6J) mice, but not resistant C57BL/6J mice. These findings suggest that *Dusp3* and *Psme3* contribute to *S. aureus* infection susceptibility in A/J mice and play a role in human *S. aureus* infection.

## Introduction

*Staphylococcus aureus* is an important cause of potentially lethal human infections [1]–[3]. It is generally accepted that host genetic variation influences susceptibility to *S. aureus* colonization and infection [4], [5].

A significant body of evidence supports the importance of human genetic variation on host susceptibility to a variety of infectious diseases. For example, TNF gene SNP rs1800629 is strongly associated with susceptibility to severe sepsis in the Chinese Han population [6], while genetic variants in TRAF6 are significantly associated with susceptibility to sepsis-induced acute lung injury [7]. In addition, a genetic variant of β2-adrenocepter gene increases susceptibility to bacterial meningitis [8], while genetic variations in Toll-like receptors have been linked with both infectious and autoimmune diseases [9]. More interestingly, genetic variation of IL17A gene is associated with altered susceptibility to Gram-positive infection and mortality of severe sepsis [10].

Far less is known about the specific genes associated with host susceptibility to *S. aureus*. Our group [11], [12] and others [5], [13] have shown that different inbred mouse strains exhibit variable susceptibility to *S. aureus* infection. For example, A/J is highly susceptible to *S. aureus* infection, whereas C57BL/6J is resistant [5]. These susceptible and resistant strains provide an attractive approach to investigate the host genetic determinants of susceptibility to *S. aureus* infection.

Using A/J donor to C57BL/6J host chromosomal substitution strains (CSS) we recently discovered that chromosomes 8, 11, and 18 from A/J account for its high susceptibility to *S. aureus* infection [11]. However, the genes on chromosome 11 that influence susceptibility to *S. aureus* remain unknown.

In the present investigation, we used a multi-step selection process to identify genes on A/J chromosome 11 contributing to susceptibility to *S. aureus* infection. Because human and murine response to sepsis can differ significantly [14], we used whole blood gene expression data from a cohort of patients with *S. aureus* blood stream infection (BSI) to verify the potential biological relevance of all candidate genes identified in mice. Genes shown to be involved in host response to *S. aureus* in both mice and humans were evaluated for biological function. Using this cross-species validation approach, we identified *Dusp3* and *Psme3* as relevant in both human and murine inflammatory response to *S. aureus* infection, and demonstrated these genes were involved in NF-κB signaling.

## Results

### Susceptibility to *S. aureus* localizes to A/J Chromosome 11

**Figure 1:**
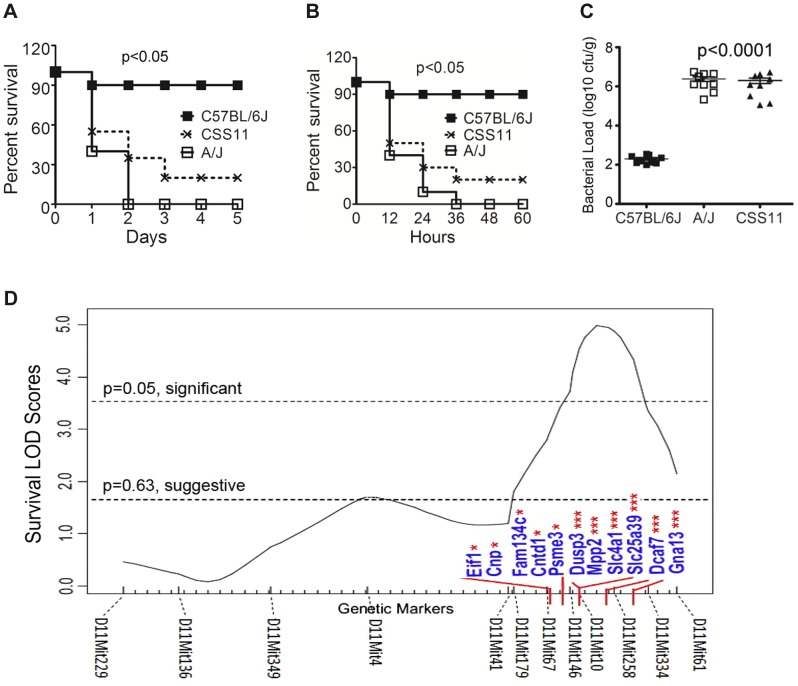
Chromosome substitution strain 11 was susceptible to *S. aureus* infection, and QTL mapping found eleven putative candidate genes on Chr11. (**A**) CSS11 was susceptible to *S. aureus* peritoneal sepsis. C57BL/6J, A/J, or CSS11 mice were injected (i.p.) with *S. aureus* Sanger 476 at 10^7^ CFU/g (n = 10 for each strain). Comparison of survival curves was performed by Mann-Whitney *u* test. The difference between C57BL/6J and CSS11 mice was significant (p<0.05). (B) CSS11 was susceptible to *S. aureus* intravenous sepsis. C57BL/6J, A/J, or CSS11 were intravenous injected with *S. aureus* Sanger 476 at 2×10^6^ CFU/g (n = 10 for each strain). Comparison of survival curves was performed by Mann-Whitney *u* test. The difference between C57BL/6J and CSS11 mice was significant (p<0.05). (**C**) Bacterial load in kidneys were significantly higher in CSS11 mice after *S. aureus* injection. C57BL/6J, A/J and CSS11 were injected (i.p.) with *S. aureus* Sanger 476 at 10^7^ CFU/g and euthanized 24 hours post infection (n = 10 for each group). The bacterial load in CSS11 kidneys were significantly higher than C57BL/6J (2.0±1.32×10^6^ CFU/ml vs 200±158 CFU/ml, p<0.0001). (**D**) Chromosome 11 LOD score plot for susceptibility to *S. aureus* in F2 intercross mice (F1 [C11A]×F1 [C11A]). Six to eight-week-old intercross mice were injected i.p. with 10^7^ CFU/g *S. aureus* Sanger 476 and observed every 8 hours continuously for 5 days. Thresholds for significant (p = 0.05) and suggestive (p = 0.63) linkage are indicated by the horizontal dashed lines. LOD score was determined by the J/qtl permutation test using 1,000 permuted data sets. The microsatellite markers for determining genotypes of F2 intercross mice are marked along the X-axis. The differentially expressed genes are indicated. Genes identified within significant or suggestive QTL were indicated with *** or *, respectively. **Footnote:** **Figure 1A and 1C** have been presented in Ahn S, et al., 2010 *PLoS Pathogens*, e1001088.

In the peritoneal *S. aureus* sepsis experiment, C57BL/6J, A/J and Chromosomal Substitution Strain 11 (CSS11) (A/J chromosome 11 in C57BL6/J background) mice were infected with *S. aureus* by an intraperitoneal (IP) route. Survival was observed for five days. C57BL/6J mice were resistant to *S. aureus* sepsis (median survival >5 days), while CSS11 mice demonstrated a susceptible phenotype (median survival <2 days) (Figure 1A) (p<0.05). In the intravenous *S. aureus* sepsis experiment, C57BL/6J, A/J and CSS11 mice were infected by direct inoculation of *S. aureus* by tail-vein route. Survival was observed every 6 hours, both A/J and CSS11 were susceptible to *S. aureus* infection (median survival <24 hours, p<0.05) as compared with C57BL/6J (Figure 1B).

The kidney bacterial load in CSS11 was also significantly higher after *S. aureus* challenge as compared with C57BL/6J (2.0±1.32×10^6^ CFU/ml vs 200±158 CFU/ml, p<0.0001) (Figure 1C), indicating the higher mortality of CSS11 strain was associated with higher *S. aureus* burden in tissue.

C57BL/6J, A/J, and CSS11 mice were also infected with *Escherichia coli* by IP injection. Survival was observed every 6 hours. Both A/J and CSS11 mice were susceptible to *E. coli* sepsis as compared with C57BL/6J (median survival <2 days; p<0.05) (Figure S1).

### Quantitative Trait Loci (QTL) mapping identifies locus on chromosome 11 linked to susceptibility to *S. aureus*

To localize the determinants on A/J chromosome 11 that are responsible for susceptibility to *S. aureus* infection, QTL analysis was performed. Previously, we established that the traits conferring susceptibility to *S. aureus* on A/J chromosome 11 were transmitted in an autosomal recessive fashion [11]. Thus, a total of 208 F2 intercross mice were generated by mating F1 (C11A) to F1 (C11A) and infected with methicillin susceptible *S. aureus* strain Sanger 476. Survival times were analyzed by J/qtl software (Jackson Labs). One significant QTL region containing 422 genes and located between 97006867 and 110713165 was significantly linked to susceptibility to *S. aureus* infection (Figure 1D).

### Microarray gene expression data identifies genes within the QTL region that are associated with susceptibility to *S. aureus* infection

**Figure 2:**
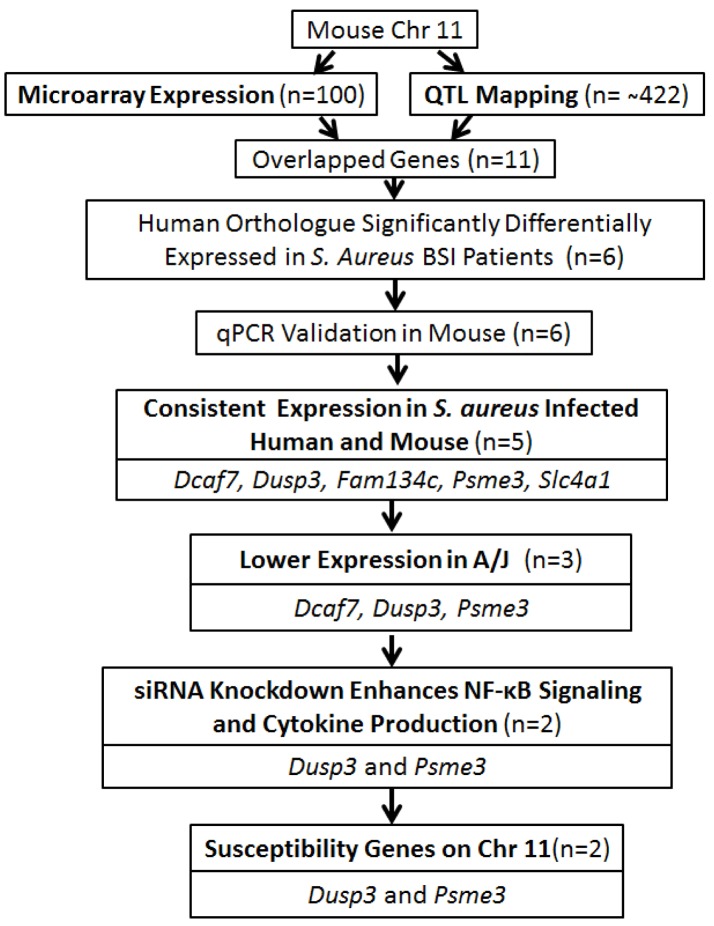
Overall strategy for identifying genes associated with *S. aureus* susceptibility on chromosome 11 of A/J mice. Flow chart of the strategy for identifying *S. aureus* susceptible genes on chromosome 11 of A/J mice.

Next, we employed the previous murine microarray expression data to further identify candidate genes located within the identified QTL region [11] (Figure 2). Genes within the identified QTL region that were differentially expressed between susceptible A/J and resistant C57BL/6J at all pre-infection and post-infection time points were considered to be putative candidate genes. A total of 11 genes met these criteria: *Eif1*, *Cnp*, *Fam134c*, *Cntd1*, *Psme3*, *Dusp3*, *Mpp2*, *Slc4a1*, *Slc25a39*, *Dcaf7*, and *Gna13* (Figure 1D). The accession numbers for each gene was listed (Table 1).

**Table 1:** Accession numbers.

Genes	Gene ID from NCBI (murine)	Gene ID from NCBI (human)
*Cnp*	12799	1267
*Cntd1*	68107	124817
*Dcaf7*	71833	10238
*Dusp3*	72349	1845
*Eif1*	20918	10209
*Fam134c*	67998	162427
*Gna13*	14674	10672
*Mpp2*	50997	4355
*Psme3*	19192	10197
*Slc4a1*	20533	6521
*Slc25a39*	68066	51629

### Consistent expression pattern between mouse candidate genes and human orthologues

**Figure 3:**
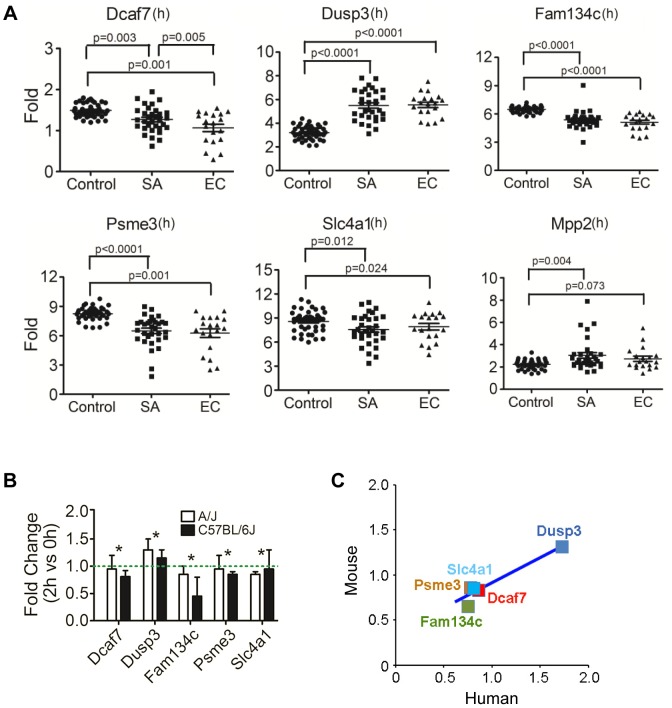
Human orthologues of six candidate genes were significantly differentially expressed between patients with *S. aureus* blood stream infection (BSI) and healthy subjects, and five of these genes (*Dcaf7*, *Dusp3*, *Fam134c*, *Psme3*, and *Slc4a1*) demonstrated consistent uninfected vs infected expression patterns between mouse and human. (**A**) Human orthologues of six candidate genes (*Dcaf7*, *Dusp3*, *Fam134c*, *Psme3*, *Slc4a1*, and *Mpp2*) were significantly differentially expressed between patients with *S. aureus* BSI and healthy subjects by microarray. Human blood RNA from patients with *S. aureus* BSI (n = 32) and healthy subjects with no infection (n = 43) were extracted and analyzed and applied to microarray. The expression of *Dusp3*(1.73 fold; p<0.0001) and *Mpp2* (1.21fold; p = 0.004) were significantly higher in *S. aureus* BSI patients as compared with healthy controls, and the expression of *Dcaf7*(0.85fold; p = 0.003), *Fam134c*(0.75fold; p<0.0001), *Psme3*(0.78fold; p<0.0001), and *Slc4a1*(*0.81fold*; p = 0.012) were significantly lower in *S. aureus* BSI patients. Six genes showed similar significant expression changes in *E. coli* BSI (n = 19) patients as compared with healthy subjects (n = 43) (**B**) Quantitative-PCR validation of the six genes identified five candidate genes (*Dcaf7*, *Dusp3*, *Fam134c*, *Psme3* and *Slc4a*) with consistent uninfected vs infected expression patterns between mouse and human. *Dcaf7* (0.83fold), *Dusp3*(1.31fold), *Fam134c* (0.65fold), *Psme3* (0.86fold), and *Slc4a1*(0.85fold). Both eight-week-old male A/J and C57BL/6J mice were i.p. injected with *S. aureus* Sanger 476 at 10^7^ CFU/g (n = 6 for each strain), at two hours post infection all mice were sacrificed by CO_2_ inhalation and whole blood were obtained through cardiac puncture. Blood RNA were extracted by QIAGEN RNeasy Protect Animal Blood Kit, and then subjected to reverse-transcription PCR and SYBR-green quantitative-PCR. The expression of all target genes were normalized to 18s rRNA. (**C**) Scatter plot of fold changes of the five candidate genes showed a consistent pattern between mouse and human. For human data analysis, the expression level of the non-infection healthy controls was set to 1, and the expression level of *S. aureus* BSI patients was normalized to the non-infection level to get the fold change. For mouse data, the fold changes were average between A/J and C57BL/6J mice.

To further evaluate the relevance of our candidate genes in human staphylococcal disease, we next used expression data from patients with *S. aureus* BSI (n = 32) or *Escherichia coli* BSI (n = 19) to evaluate the potential clinical relevance of our identified candidate genes. Among the 11 putative genes identified in the murine model, six were found to have human orthologues that exhibited significantly different levels of expression in patients with *S. aureus* BSI as compared to healthy subjects with no infection (n = 43): *Dcaf7* (0.85fold; p = 0.003), *Dusp3* (1.73 fold; p<0.0001), *Fam134c* (0.75fold; p<0.0001), *Psme3* (0.78fold; p<0.0001), *Mpp2* (1.21fold; p = 0.004), and *Slc4a1* (*0.81fold*; p = 0.012) (Figure 3A). The gene expression patterns for the six genes were also significantly different among the patients with *E. coli* BSI vs. healthy subjects (Figure 3A).

Using qPCR on the murine samples, five of the 6 genes identified by both human and murine gene expression also exhibited consistent expression changes under infectious vs. non-infectious conditions : *Dcaf7* (0.83fold), *Dusp3* (1.31fold), *Fam134c* (0.65fold), *Psme3* (0.86fold), and *Slc4a1*(0.85fold) (Figure 3B). The expression patterns for these five genes were highly consistent between *S. aureus*-infected mice and *S. aureus*-infected humans (Figure 3C). The expression patterns of these five genes also remained consistent when mice were infected with *E. coli* instead of *S. aureus* (Figure S2). qPCR primers for candidate genes were listed (Table S1).

### qPCR of candidate genes

**Figure 4:**
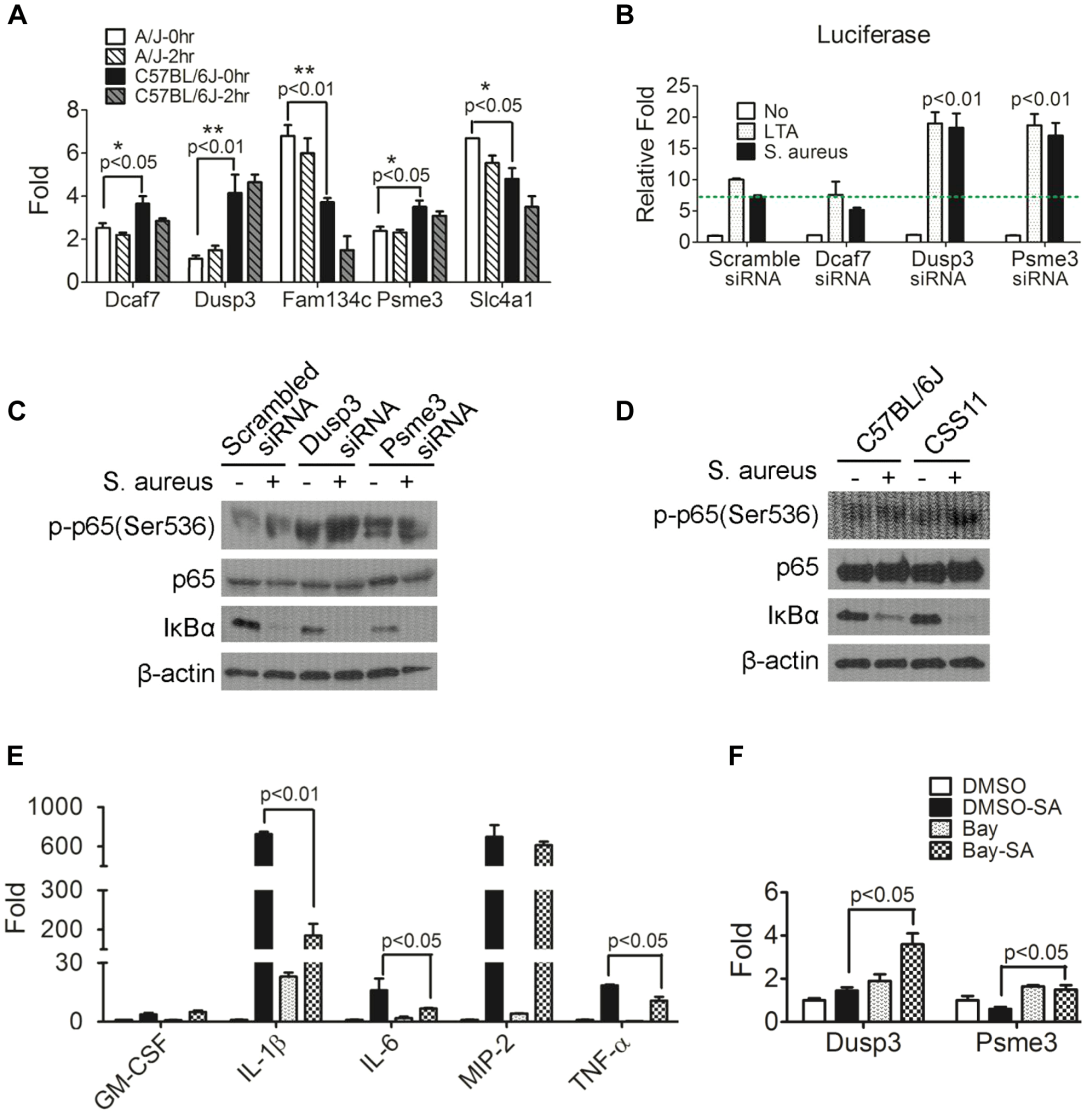
Down-regulation of *Dusp3* and *Psme3* in A/J are responsible for increased NF-κB signaling activity. (**A**) The expression of *Dcaf7*, *Dusp3*, and *Psme3* in A/J was significantly lower than C57BL/6J under both non-infected and *S. aureus* infected conditions. Eight-week-old male A/J and C57BL/6J mice (n = 6) were challenged (i.p.) by *S. aureus* Sanger 476 at 10^7^ CFU/g or DPBS. At two hours post-infection whole blood RNAs was extracted by RNeasy followed by RT-PCR and qPCR. *Dcaf7* (0.81 fold; p<0.05), *Dusp3* (0.27 fold; p<0.01), and *Psme3* (0.83 fold; p<0.05) were down-regulated in A/J mice at baseline (0 hr) as compared with resistant C57BL/6J. Two genes exhibited elevated expression in susceptible A/J mice (baseline): *Fam134c* (1.82 fold; p<0.01) and *Slc4a1* (1.31fold; p<0.05). The baseline difference in expression between susceptible (A/J) and resistant (C57BL/6J) mice of all the five genes remained unchanged at 2 hr post *S. aureus*-infection. (**B**) *Dusp3* and *Psme3* inhibit NF-κB signaling activity in RAW264.7 macrophages. RAW264.7 cells co-transfected with NF-κB-luciferase and pRL-TK plasmids were then transfected with siRNA of each individual candidate genes (*Dcaf7*, *Dusp3*, *Psme3*) or scrambled siRNA. Then transfected RAW cells were stimulated by either medium alone, medium containing LTA (10 µg/ml) or medium containing *S. aureus* particles (10 µg/ml) for 7 hours. Cells were directly lysed by 1× passive lysis buffer and luciferase activity was assayed and normalized to renilla activity as previously described [15]. As shown, knockdown of both *Dusp3* and *Psme3* significantly up-regulates NF-κB luciferase activity (p<0.01). (**C**) Knockdown of *Dusp3* or *Psme3* enhanced the activation of NF-κB signaling upon *S. aureus* stimulation. BMDMs from C57BL/6J were transfected with either scrambled, *Dusp3* or *Psme3* siRNA, then stimulated with *S. aureus* for 15 minutes. Whole cell lysate was loaded for western-blot. Knockdown of *Dusp3* or *Psme3* dramatically increased degradation of IκBα and phosphorylation of p65 (Ser536) as compared with scrambled siRNA control. (**D**) Enhanced NF-κB signaling upon *S. aureus* stimulation in CSS11 BMDMs. BMDMs from either C57BL/6J or CSS11 were stimulated with *S. aureus* for 15 minutes. Whole cell lysate was loaded for Western blot. BMDMs from CSS11 exhibited increased degradation of IκBα and phosphorylation of p65 (Ser536) as compared with BMDMs from C57BL/6J. (**E**) Bay inhibition of NF-κB dramatically suppressed cytokine production. RAW264.7 macrophages were pre-treated with 4 µM Bay 11-7085 for one hour, then stimulated with 10 µg/ml *S. aureus* particles in 2 µM Bay for 3 hours. RNA was extracted and subjected to reverse-transcription PCR and qPCR. Inhibition of NF-κB by Bay inhibitor dramatically suppressed cytokine production upon *S. aureus* stimulation, including IL-1β (p<0.01), IL-6 (p<0.05) and TNF-α (p<0.05). (**F**) Inhibition of NF-κB enhanced *Dusp3* and *Psme3* expression. The inhibition of NF-κB activity by Bay inhibitor significantly enhanced the expression of both *Dusp3* (p<0.05) and *Psme3* (p<0.05), which indicated a reciprocal relationship between NF-κB signaling activity and *Dusp3* or *Psme3* expression.

Using qPCR in susceptible (A/J) vs resistant (C57BL/6J) mice at baseline (0 hr), three of the five genes demonstrated significantly lower expression: *Dcaf7* (0.81 fold; p<0.05), *Dusp3* (0.27 fold; p<0.01), and *Psme3* (0.83 fold; p<0.05), while two genes exhibited significantly higher expression: *Fam134c* (1.82 fold; p<0.01), *Slc4a1* (1.31fold; p<0.05) (Figure 4A). The baseline difference in expression between susceptible (A/J) and resistant (C57BL/6J) mice for all the five genes remained unchanged at 2 hr post-infection of *S. aureus* (Figure 4A).

### Candidate genes influence NF-κB signaling pathway

Since NF-κB signaling plays a crucial rule in host defense to various pathogens [15]–[17] including *S. aureus* [18], [19], the impact of the five putative genes on NF-κB signaling was analyzed by co-transfection of NF-κB-luciferase reporter plasmid and siRNA of each gene into RAW264.7 murine macrophage cell line. The siRNAs used in this study were listed (Table S2). The siRNA of each candidate gene efficiently reduced gene expression in RAW 264.7 cells (Figure S3). Knockdown of two genes, *Dusp3* (p<0.01) and *Psme3* (p<0.01), significantly enhanced NF-κB signaling upon stimulation with either lipoteichoic acid (LTA) or *S. aureus* particles (Figure 4B). Because both of these genes (*Dusp3* and *Psme3*) were down-regulated in A/J at baseline and *S. aureus* infection (Figure 4A), siRNA-mediated knockdown mimicked the status of susceptible A/J in RAW264.7 murine macrophages. These results suggest that lower expression of *Dusp3* and *Psme3* in A/J mice could explain the observed susceptibility to *S. aureus*.

To better understand how *Dusp3* and *Psme3* affect NF-κB signaling activity, phosphorylation of p65 at Ser536 and degradation of IκBα were analyzed by western blot. BMDMs from C57BL/6J were transfected with either scrambled, *Dusp3*, or *Psme3* siRNA one day before and then subjected to *S. aureus* challenge for 15 minutes. The knock-down efficiency was tested in a parallel experiment (Figure S4). Western blot results showed that knockdown of *Dusp3* or *Psme3* dramatically increased phosphorylation of p65 at Ser536 as compared with scrambled siRNA (Figure 4C, top panel). The degradation of IκBα was also increased in either *Dusp3* or *Psme3* knockdown cells (Figure 4C, second bottom panel). The antibodies used in this study were listed (Table S3).

Similarly, BMDMs from CSS11 exhibited increased phosphorylation of p65 (Ser536) and degradation of IκBα after *S. aureus* stimulation as compared with BMDMs from C57BL/6J (Figure 4D). These data indicate that the suppressive function of *Dusp3* and *Psme3* on NF-κB signaling happens prior to the inhibitory cytosolic complex of IκBα-p65-p50. The signaling event should happen within the cytosol or at the cell membrane rather than in the nucleus, and probably in the proximal signaling stage before IκBα degradation.

### NF-κB signaling is responsible for inflammatory cytokine production

Next, we analyzed cytokine and chemokine production after inhibiting NF-κB signaling. Either Bay 11-7085 or DMSO was applied to RAW264.7 cells before *S. aureus* challenge. qPCR illustrated that Bay inhibition of NF-κB signaling significantly suppressed inflammatory cytokine and chemokine production upon *S. aureus* stimulation in RAW264.7 macrophages, including IL-1β (p<0.01), IL-6 (p<0.05), and TNF-α (p<0.05) (Figure 4E). Inhibition of NF-κB signaling also enhanced the expression of *Dusp3* (p<0.05) and *Psme3* (p<0.05), suggesting a negative feedback regulatory loop between NF-κB and both *Dusp3* and *Psme3* (Figure 4F). qPCR primers for cytokines and chemokines applied in this study were listed (Table S4).

### Suppressive regulation of cytokine production by *Dusp3* and *Psme3*

**Figure 5:**
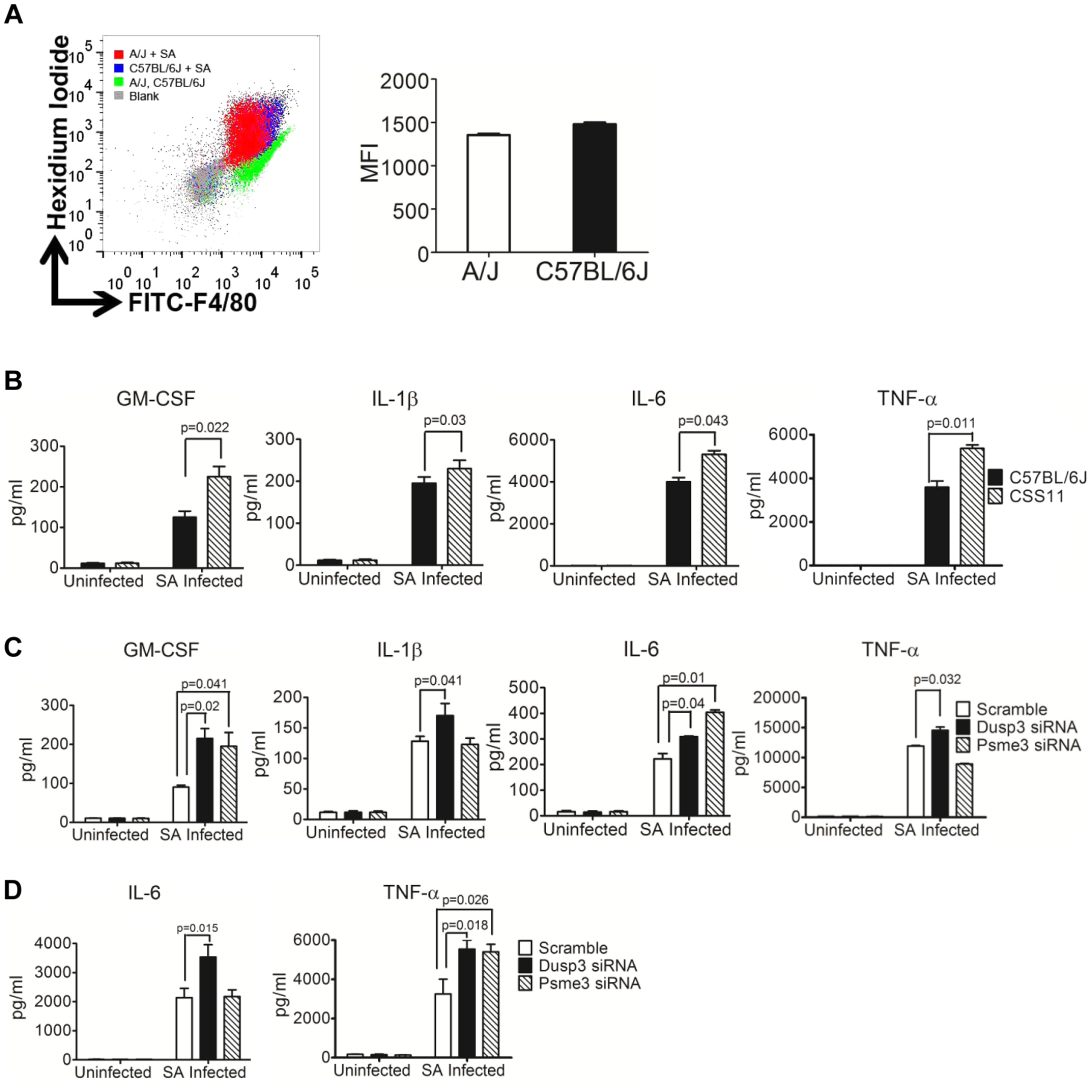
siRNA knockdown of *Dusp3* and *Psme3* result in significant elevation of cytokine production, consistent with the pattern of bone marrow derived macrophages from CSS11 as compared with C57BL/6J (GM-CSF, IL-1β, IL-6 and TNF-α). (**A**) Bone-marrow derived macrophages (BMDMs) from A/J and C57BL/6J have similar *S. aureus* phagocytosis ability. 2×10^6^ BMDMs were seeded to single wells in a 6-well plate the day before phagocytosis and incubated with hexidium iodide stained *S. aureus*. Phagocytic efficiency as determined by the mean fluorescence intensity (MFI) is not significantly different in BMDMs from C57BL/6J and CSS11 mice. Representative histogram of 3 separate experiments. (**B**) BMDMs from CSS11 mice produced significantly higher cytokine levels as compared to C57BL/6J. 4×10^5^ BMDMs from both C57BL/6J and CSS11 mice were seeded to single-wells of a 24-well plate the day before infection. Infection was simulated by adding *S. aureus* particles at 10 µg/ml. At 24 hours post-infection the supernatants were harvested and subjected to Luminex cytokine assaying. BMDMs from CSS11 mice significantly enhanced cytokine production, including GM-CSF, IL-1β, IL-6, and TNF-α. (**C**) Down-regulation of *Dusp3* and *Psme3* by siRNA led to up-regulation of cytokine production upon *S. aureus* challenge in RAW264.7 macrophages. RAW264.7 cells were transfected by either scramble or *Dusp3* or *Psme3* siRNA, and then infected with *S. aureus* particles at 10 µg/ml as before [11]. At 24 hours post-infection, the supernatants were harvested and subjected to Luminex-multiplex cytokine assaying. The down-regulation of *Dusp3* significantly enhanced cytokine production, including GM-CSF, IL-1β, IL-6, and TNF-α, as compared to scramble siRNA control. The down-regulation of *Psme3* also significantly elevated GM-CSF and IL-6 production. (**D**) Down-regulation of *Dusp3* or *Psme3* by siRNA led to up-regulation of cytokine production upon *S. aureus* challenge in BMDMs. BMDMs from C57BL/6J were transfected by either scrambled, *Dusp3* or *Psme3* siRNA, and then infected with *S. aureus* particles at 10 µg/ml. At 24 hours post-infection, the supernatants were harvested and subjected to cytokine analysis. The down-regulation of *Dusp3* significantly enhanced cytokine production, including IL-6 and TNF-α, as compared to scrambled siRNA control. The down-regulation of *Psme3* also significantly elevated TNF-α production.

Neutrophils and macrophages from A/J and C57BL/6J exhibit similar bacterial killing capacity [11], suggesting that other host characteristics account for differences in the *S. aureus* susceptibility phenotype. To evaluate these factors, we used a well-established macrophage differentiation system [15] to differentiate the bone-marrow progenitor cells from A/J and C57BL/6J into macrophages and analyzed macrophage markers by flow cytometry. No significant differences in either CD11b or F4/80 expression were observed (Figure S5). Using flow cytometry, bone marrow derived macrophages (BMDMs) from A/J and C57BL/6J exhibited a similar phagocytosis capacity for *S. aureus* (Figure 5A).

Luminex cytokine profiling showed that BMDMs from CSS11 produced more inflammatory cytokines as compared with C57BL/6J in response to stimulation with *S. aureus*, including GM-CSF (p = 0.022), IL-1β (p = 0.03), IL-6 (p = 0.043), and TNF-α (p = 0.011) (Figure 5B, and S6). This elevation in cytokine production is likely to be due to the down-regulation of *Dusp3* and *Psme3* in CSS11. siRNA knockdown of these two genes in *S. aureus*-challenged RAW264.7 macrophages and BMDMs greatly enhanced NF-κB signaling (Figure 4B and 4C), which in turn directly activated cytokine and chemokine production (Figure 4E). Indeed, in *Dusp3* siRNA transfected *S. aureus*-challenged RAW264.7 macrophages, Luminex-multiplex profiling detected significantly enhanced cytokine production as compared with scrambled siRNA, including GM-CSF (p = 0.02), IL-1β (p = 0.041), IL-6 (p = 0.04), and TNF-α (p = 0.032) (Figure 5C and S7). Similarly, *Psme3* siRNA transfected, *S. aureus*-challenged RAW264.7 cells also produced significantly more GM-CSF (p = 0.041) and IL-6 (p = 0.01) than control (Figure 5C and S7). Similarly, in *S. aureus* challenged BMDMs of C57BL/6J, knockdown of *Dusp3* dramatically enhanced the production of IL-6 (p = 0.015) and TNF-α (p = 0.018) (Figure 5D and S8). Likewise, knockdown of *Psme3* enhanced the production of TNF-α (p = 0.026) as compared with scrambled siRNA (Figure 5D and S8).

**Figure 6:**
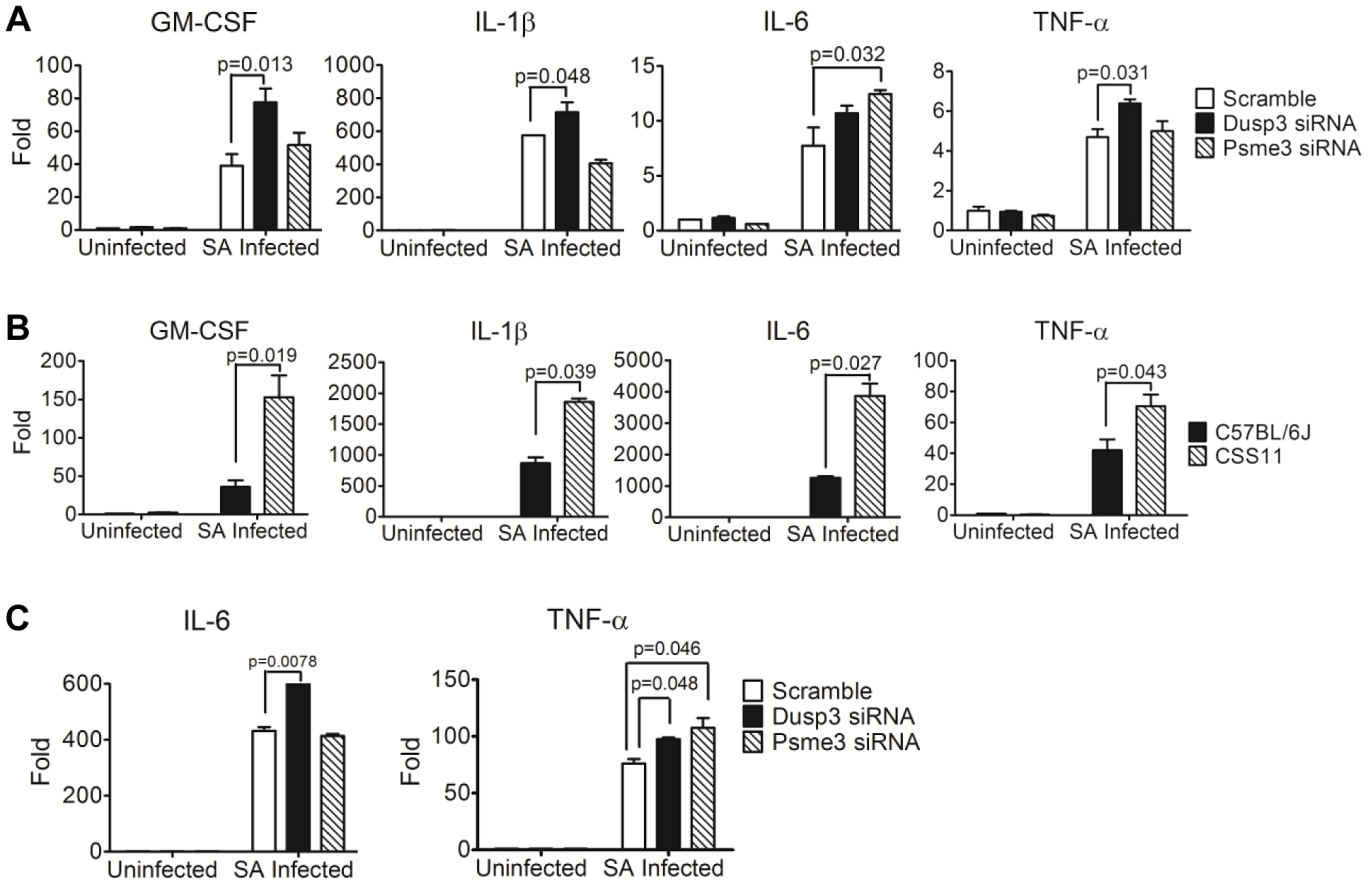
Quantitative-PCR confirmed elevation of cytokine production in macrophages transfected by *Dusp3* and *Psme3* siRNA or BMDMs from CSS11(GM-CSF, IL-1β, IL-6, and TNF-α). (**A**) Down-regulation of *Dusp3* and *Psme3* by siRNA led to increased cytokine RNA expression upon *S. aureus* challenge in RAW264.7 macrophages. At three hours post-infection total RNA was extracted followed by reverse-transcription PCR and SYBR-Green qPCR. The expression of all genes were normalized to 18s rRNA. The expression level of GM-CSF, IL-1β, IL-6, and TNF-α was higher in *Dusp3* knockdown RAW cells, and the level of GM-CSF and IL-6 was higher in *Psme3* knockdown RAW cells. p-value smaller than 0.05 was considered significant. (**B**) BMDMs cytokine RNA production in CSS11 mice was significantly higher than in C57BL/6J upon *S. aureus* infection. 2×10^6^ BMDMs were seeded to single wells in a 6-well plate the day before infection. At three hours post-infection, RNA was extracted using RNeasy followed by RT-PCR and qPCR. The expression levels of GM-CSF, IL-1β, IL-6, and TNF-α were significantly higher in BMDMs from CSS11 mice. The expression of all genes were normalized to 18s rRNA. p-value smaller than 0.05 was considered significant. (**C**) Down-regulation of *Dusp3* and *Psme3* by siRNA led to increased cytokine RNA expression upon *S. aureus* challenge in BMDMs of C57BL/6J. The expression level of IL-6 was higher in *Dusp3* siRNA transfected BMDMs, and the expression of TNF-α was higher in both *Dusp3* and *Psme3* siRNA transfected BMDMs.

Patterns of increased cytokine production were also consistent between mRNA production from *Dusp3* or *Psme3* siRNA transfected RAW 264.7 macrophages or BMDMs and primary CSS11 BMDMs. Knockdown of *Dusp3* in RAW264.7 macrophages significantly increased cytokine mRNA production upon *S. aureus* challenge as compared with scrambled control, including GM-CSF (p = 0.013), IL-1β (p = 0.048), and TNF-α (p = 0.031) (Figure 6A and S9). Knockdown of *Psme3* in RAW264.7 macrophages also significantly increased IL-6 (p = 0.032) mRNA production (Figure 6A and S9). Primary BMDMs from CSS11, which contains chr. 11 from A/J in an otherwise C57BL/6J background, expressed more cytokine mRNA upon *S. aureus* challenge as compared with C57BL/6J, including GM-CSF (p = 0.019), IL-1β (p = 0.039), IL-6 (p = 0.027), and TNF-α (p = 0.043). This observation is likely to reflect the down-regulation of both *Dusp3* and *Psme3* in the A/J chromosome 11 contained by CSS11 mouse lineage (Figure 6B and 4A). Similarly, in *S. aureus* challenged BMDMs of C57BL/6J, knockdown of *Dusp3* dramatically increased IL-6 RNA (p = 0.0078) and TNF-α RNA (p = 0.048) (Figure 6C). Knockdown of *Psme3* significantly increased TNF-α RNA (p = 0.046) as compared with scrambled siRNA (Figure 6C).

### Pre-exposure to *S. aureus* impairs phagocytosis ability

Since NF-κB signaling mediates host defenses generally in a positive way, we hypothesized that persistent, unbated stimulation of production of antimicrobial effectors from immune cells, such as antimicrobial peptides, would eventually lead to “immune paralysis” or “immune exhaustion” that impeded further defense against *S. aureus* challenge. To test this hypothesis, BMDMs from both C57BL/6J and A/J were pre-exposed to either TNF-α (100 ng/ml) or *S. aureus* particles (10 µg/ml), then subjected to phagocytosis analysis. Pre-exposure to TNF-α enhanced the phagocytosis capacity of BMDMs from both strains (Figure S10). However, pre-exposure to *S. aureus* particles reduced phagocytosis ability of BMDMs from both strains, and the reduction was more extensively in BMDMs from A/J (Figure S10). These results suggest that prolonged simultaneous activation of all pathways in the host by *S. aureus*, and not isolated stimulation of the TNFα-TNF receptor pathway alone, impaired the immune system's ability to further respond to *S. aureus* infection.

### Expression of *Dusp3* and *Psme3* in human neutrophils and macrophages with *S. aureus* infection

Next, we analyzed the role of *Dusp3* and *Psme3* upon *S. aureus* challenge in the immortalized human monocyte cell line U-937. Despite efficient knock-down of *Dusp3* and *Psme3*, *S. aureus* stimulation of U-937 led to cell death rather than activation of NF-κB signaling and cytokine production (data not shown). We therefore concluded that U-937 was not suitable for our current analysis, and instead evaluated function of these two genes in human samples. To do this, we used publically available datasets of various human immune cells challenged by *S. aureus*, including human neutrophils (http://www.ncbi.nlm.nih.gov/geo/query/acc.cgi?acc=GSE16837) and human macrophages (http://www.ncbi.nlm.nih.gov/geo/query/acc.cgi?acc=GSE13670). In the human neutrophil dataset (GEO:GSE16837), *Dusp3* increased to 9.88 fold at 3 hr (p<0.001) and 7.95 fold at 6 hr (p<0.05) as compared with 0 hr after *S. aureus* stimulation; and *Psme3* decreased to 0.68 fold at 3 hr (p<0.0001) and 0.45 fold at 6 hr (p<0.0001) as compared with 0 hr (Figure S11). In the human macrophage dataset (GEO:GSE13670), *Dusp3* increased to 1.62 fold at 8 hr following *S. aureus* challenge compared with controls (p<0.005) and *Psme3* decreased to 0.73 fold at 8 hr following *S. aureus* challenge as compared with each control (p<0.001) (Figure S11). Collectively, these data and our human and murine data together strongly support that *Dusp3* and *Psme3* are candidate genes highly associated with *S. aureus* susceptibility.

## Discussion

**Figure 7:**
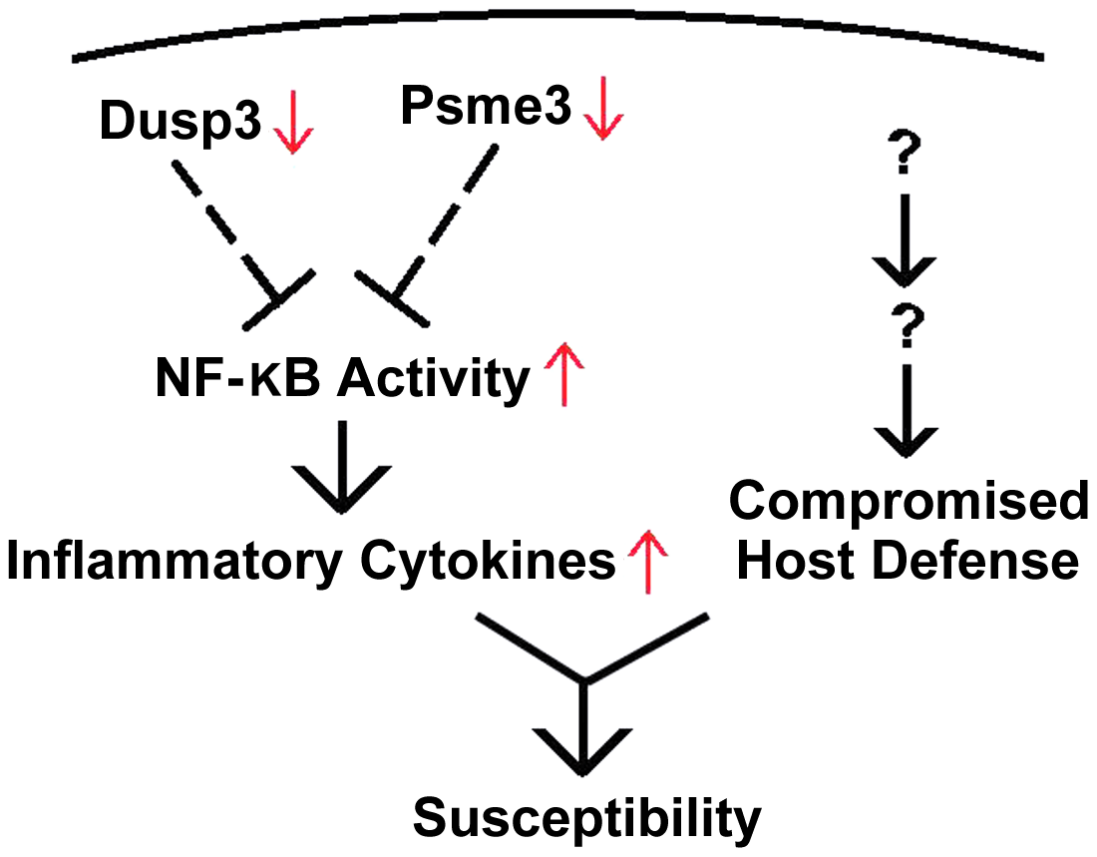
Down-regulation of *Dusp3* and *Psme3* in A/J is associated with over-production of pro-inflammatory cytokines. Both *Dusp3* and *Psme3* inhibit NF-κB activity, which is responsible for the production of inflammatory cytokines. The reduced expression of *Dusp3* and *Psme3* in A/J mice is associated with elevated NF-κB activity, which leads to increased inflammatory cytokines. *Dusp3* and *Psme3*, together with other factors from murine chromosome 8 and 18, contribute to *S. aureus* susceptibility of A/J strain.

The genetic basis for host susceptibility to *S. aureus* is largely unknown. In the current report, we have identified two genes, *Dusp3* and *Psme3*, which are strongly associated with *S. aureus* sepsis in mice and humans, and have proposed a potential biological mechanism as negative feedback components in NF-κB-mediated signaling (Figure 7). These factors are likely to contribute to host susceptibility to *S. aureus* sepsis in mouse, and potentially in humans.

We identified a QTL locus on chromosome 11 that was significantly linked to *S. aureus* susceptibility in A/J mice. We found five candidate genes (*Dcaf7*, *Dusp3*, *Fam134c*, *Psme3*, and *Slc4a1*) within the QTL locus that were differentially expressed between susceptible and resistant mouse strains and had human orthologues with the same significant expression patterns in patients with both *S. aureus* BSI and *E. coli* BSI. The results suggest that one or more of these five genes are involved in a common host response to *S. aureus* infection in both humans and mice. qPCR demonstrated that two of these five genes, *Dusp3* and *Psme3*, were down-regulated in susceptible vs. resistant mice, and exhibited a significantly enhanced inflammatory cytokine response when knocked down with siRNA in both RAW264.7 macrophages and BMDMs. The over-production of inflammatory cytokines encountered with siRNA-mediated knockdown of these two genes was consistent with that encountered in *S. aureus*-challenged bone marrow derived macrophages from CSS 11 (C57BL/6J background with A/J chromosome 11) mice. Taken together, these data provide strong evidence that *Dusp3* and *Psme3* may be key genes involved in the host susceptibility to *S. aureus*.

Previous investigations have shown inconsistencies between murine and human response to sepsis [20]–[23]. For example, a recent study by Seok and colleagues demonstrated significant differences between murine and human genomic responses to several acute inflammatory diseases, including burns, trauma, and endotoxemia [14]. Our candidate gene selection approach ensured that all murine candidate genes were also relevant in humans with *S. aureus* BSI. In this way, almost two-thirds of the more than 1000 genes on A/J chromosome 11 were excluded from contributing to susceptibility to *S. aureus*. Using this trans-species comparative genomic approach, only *Dusp3* and *Psme3* were identified as putative contributors to *S. aureus* susceptibility and both exhibited a significant biological effect on NF-κB signaling. Because NF-κB is centrally involved in the inflammatory response in both mouse and human [24], [25], these two genes are likely to be involved in the host inflammatory response to *S. aureus* in both humans and mice.

The diverse function of *Dusp3* requires further detailed investigation on its biological relevance to *S. aureus* susceptibility. First, it is greatly involved in signaling transduction pathways regulating protein de-phosphorylation. For example, *Dusp3* has been reported to be the main protein tyrosine phosphatase in macrophage mediating cellular processes, including immune response [26]. It is a redox-sensitive and ERK-specific phosphatase. Bacterial colonization results in oxidative inactivation of *Dusp3* and consequently stimulation of ERK [27]. *Dusp3* also regulates cell death and cell proliferation, exhibiting anti-apoptotic ability in prostate cancer cells and promoting cell cycle progression in carcinoma of the cervix [28], [29]. Perhaps most interestingly, *Dusp3* has been shown to affect the expression of vascular endothelial growth factor (VEGF) [30]–[32]. VEGF has been reported to increase cardiovascular collapse and vascular permeability during *S. aureus* sepsis pathogenesis and has been proposed as a major determinant of vascular hyperpermeability in MRSA sepsis [30], [31]. In our experiment, VEGF was also significantly elevated in *Dusp3* siRNA transfected RAW264.7 macrophages in both non-infection and *S. aureus* infection condition. Finally, *Dusp3* homozygous mutant mice exhibited susceptibility to Gram negative bacterial infection in screening results from the Knockout Mouse Consortium program [33], [34]. Collectively, this evidence suggests the *Dusp3* pathway may be an important candidate pathway in resolving *S. aureus* related pathogenesis.

*Psme3* is a subunit of a proteasome responsible for the generation of peptides loaded onto MHC class I molecules [35], [36]. MHC class I molecules are crucial components of host response to infection, contributing to host recognition of viral and bacterial infected cells by host cytotoxic CD8-T cells for the final killing and degradation of pathogens [37], [38]. The down-regulation of *Psme3* in A/J but not C57BL/6J may result in less degradation of phagocytosed *S. aureus*, less *S. aureus* antigen presented on MHC class I molecules, and ultimately less degradation of infected host cells by cytotoxic CD8-T cells. Interestingly, siRNA-mediated down-regulation of *Psme3* also dramatically increased VEGF production by RAW264.7, further suggesting that VEGF enrichment affected by down-regulation of *Dusp3* and *Psme3* in murine chromosome 11 contributes to the *S. aureus* susceptible phenotype of A/J.

In the process of sepsis, harmful molecular mechanisms contribute to the high mortality seen in severely ill patients [39], [40]. In these cases, a pathogenic role of excessive immunity, also known as “cytokine storm” and reduced immunity through immune paralysis are highly associated with death induced by acute bacterial infection. Referred to as “compensatory anti-inflammatory response syndrome” (CARS), this “immune-exhaustion” phenomenon is the consequence of counter-regulatory mechanisms initiated to limit the over-activated inflammatory response in sepsis patients [41], [42]. In patients with CARS, over-activation of inflammatory response ultimately leads to changes in expression of genes associated with phagocytosis, antigen presentation, cell migration and apoptosis [43]. In the current investigation, downregulation of *Dusp3* and *Psme3* in A/J increased the production of pro-inflammatory cytokines, which may partially account for the cytokine storm observed in that mouse lineage when infected with *S. aureus*. These two genes, combined with other factors from chromosome 8 and 18, would together contribute to susceptibility to *S. aureus* in A/J mice, and potentially in humans.

Further, our studies showed that pre-exposure of macrophages to *S. aureus* particles compromised their ability to further take up the bacteria. This finding suggests that persistent, unabated stimulation of the immune system by *S. aureus* infection can eventually lead to immune paralysis or exhaustion of antimicrobial peptides. Since the overproduction of cytokines is the major phenotype of CSS11, we hypothesize that cytokine storm could account for the increased susceptibility of A/J to dying of *S. aureus* sepsis. Given these findings, we hypothesize that down-regulation of *Dusp3* and *Psme3* in A/J result in hyper-responsiveness of host immune system, which in turn leads to “immune paralysis” of the host to further defense against prolonged *S. aureus* challenge. Collectively, these finding also indicate that immediate bacterial clearance is important for host defense against *S. aureus*.

Several of the other genes identified in our experiments were also promising candidates. *Dcaf7* is a scaffold protein for activating MEKK1 kinase [44] and is involved in the human TNFα/NF-κB signal transduction pathway [45]. A dominant negative mutant of MEKK1 was reported to abolish T-cell receptor activation by super-antigen staphylococcus enterotoxin E [46] while NF-κB mediated innate immune defense against *S. aureus* through TLR2 and NOD2 [16], [47], [48]. *Fam134c* is a family with sequence similarity to 134, member C. Little is known of the putative function of *Fam134c* or related family members [49]. However, mice with *Fam134c* homozygous mutations have demonstrated bacterial susceptible phenotype based on the screening results from the Knockout Mouse Consortium program [33], [34]. *Slc4a1* is a membrane protein or protein of membrane related organelles mediating small molecule transporting and intracellular metabolism [50]–[52].

The current study has limitations. First, our cohort of patients did not include subjects who were colonized, but not infected, with *S. aureus*. Second, the two model systems used in this manuscript (intraperitoneal sepsis, tail vein sepsis) fail to fully represent the diversity of human infections caused by *S. aureus* (e.g., endocarditis, osteomyelitis, visceral abscesses, pneumonia, soft tissue infection). Our approach does not consider the impact of post-translational modification [53], [54] and single nucleotide polymorphisms on genes and their products. Thus, there may be additional candidate genes on chromosome 11 beyond *Dusp3* and *Psme3*. Moreover, our approach may have missed genes that contribute to the susceptible phenotype by way of a joint or additive effect. In support of this possibility is the fact that our original discovery found that three chromosomes, 8, 11, and 18, were each independently associated with susceptibility to *S. aureus* [11]. Thus, additional experiments are underway in our lab, including QTL mapping analysis for A/J chromosome 8, defining the pathogenesis of *Dusp3* and *Psme3* using knockout mice, and evaluating the impact of *Dusp3* and *Psme3* on host susceptibility to different pathogens.

Despite these limitations, this study makes several key observations. First, we have identified one QTL on chromosome 11 that is significantly linked to survival time after infection with *S. aureus*. Eleven differentially expressed genes mapped to the significant- or suggestive- threshold of this QTL. Five of these 11 genes exhibited significant evidence of involvement in patients with *S. aureus* BSI that was consistent with the pattern encountered in the murine model. Two of these five genes, *Dusp3* and *Psme3*, responded to *S. aureus* challenge by negatively regulating NF-κB signaling, leading to enhanced cytokine response (GM-CSF, IL-1β, IL-6, and TNFα). Consistent with the hypothesis of enhanced A/J susceptibility caused by unchecked inflammatory response, *Dusp3* and *Psme3* were less expressed in susceptible A/J as compared with resistant C57BL/6J. All of our results support a potential role of these two genes in host response to *S. aureus*. *Dusp3* and *Psme3* represent promising candidates for the genetic basis of host susceptibility to *S. aureus*.

## Materials and Methods

### Ethics statement

All animal experiments were carried out in strict accordance with the recommendations of NIH guidelines, the Animal Welfare Act, and US federal law. All animal procedures were approved by the Institutional Animal Care and Use Committee (IACUC Protocol A191-12-07) of Duke University which has been accredited by the Association for Assessment and Accreditation of Laboratory Animal Care (AAALAC) International. All animals were housed in a centralized and AAALAC accredited research animal facility that is fully staffed with trained husbandry, technical, and veterinary personnel. The Institutional Review Boards from all involved hospitals approved the human studies referenced in this work. Written informed consent was obtained for all subjects after the nature and possible consequences of the studies were explained.

### Human subjects

Subjects were enrolled at Duke University Medical Center (DUMC; Durham, NC), Durham VAMC (Durham, NC), and Henry Ford Hospital (Detroit, Michigan) as part of a prospective, NIH-sponsored study to develop novel diagnostic tests for severe sepsis and community-acquired pneumonia as mentioned before [55]–[57]. All participants were adults. RNA was obtained from blood drawn at the time patients initially presented to the Emergency Department with sepsis. RNA expression data from patients who were ultimately found to have BSI with either *S. aureus* (n = 32) or *E. coli* (n = 19) were used in this study. Healthy controls were defined as uninfected human (n = 43), enrolled as part of a study on the effect of aspirin on platelet function among healthy volunteers. Subjects were recruited through advertisements posted on the Duke campus. Blood used to derive gene expression data in these healthy controls was drawn prior to aspirin challenge. Human orthologs of murine genes were identified by Chip comparer (http://chipcomparer.genome.duke.edu/) as reported before [58]. When there were multiple orthologs, we preferentially used the anti-sense target probes that shared the fewest probes with other genes as identified by the probe label.

### Mouse strains

C57BL/6J, A/J, and CSS11 mice were purchased from the Jackson Laboratory (Bar Harbor, ME). All the mice were allowed to acclimate for more than 7 days before experiments. For generation of F1 progeny, CSS11 mice were mated with C57BL/6J in reciprocal crosses [C57BL/6J male×CSS11 female and C57BL/6J female×CSS11 male] to generate an F1 population with heterozygous chromosome 11 due to homologous recombination. To generate F2 intercross mice for QTL linkage analysis, F1 (C11A) mice were intercrossed with F1 (C11A) to produce more than 200 progeny.

### Preparation of bacteria

*S. aureus* clinical strain, Sanger 476 was used in the mortality and infection studies. For preparation of *S. aureus* for injection, overnight culture of *S. aureus* was diluted 100 folds with fresh tryptic soy broth (TSB) and shake at 37°C with aeration to log-phase (OD600≈0.8). *S. aureus* was harvested by centrifugation at 3000 rpm for 10 minutes at 4°C, washed once in DPBS and re-suspended in DPBS.

### Murine sepsis experiment and bacterial load quantification

For murine peritonitis-sepsis experiments, 8-week-old male mice (n = 10) in each strain of C57BL/6J, A/J, and CSS11 were i.p. injected with 10^7^ CFU/g *S. aureus* (Sanger 476) or 2×10^5^ CFU/g *E. coli* (K1H7) and observed every 6 hours for morbidity continuously for 5 days. For murine intravenous sepsis, 8-week-old male mice (n = 10) in each strain of C57BL/6J, A/J, and CSS11 were i.v. injected with 2×10^6^ CFU/g *S. aureus* (Sanger 476) and observed every 6 hours for morbidity continuously for 3 days. For bacterial load quantification, kidneys were collected from euthanized mice at 24 hours post-infection, then homogenized in DPBS and serially diluted (10 fold). The dilutions were plated in Tryptic Soy Agar (TSA) plates and incubated at 37°C overnight for counting colony forming units (CFU).

### QTL linkage analysis

Polymorphic microsatellite markers on chromosome 11 between C57BL/6J and A/J were chosen from a database maintained by Mouse Genomic Informatics (http://www.informatics.jax.org/). Twelve microsatellite markers were selected with an average inter-marker distance of 3.1 cM covering chromosome 11. A total of 208 F2 intercross were generated, all of which were genotyped for each microsatellite marker by PCR amplification and gel electrophoresis. J/qtl software was used to analyze phenotype and genotype data for linkage of survival time after infection with *S. aureus* Sanger 476 and marker location. Phenotypes were defined as either sensitive or resistant based on the dichotomization of survival data (survival of less than 2 day is “0” and survival of longer than 2 days is “1”, respectively). All linkage analysis results were expressed as LOD scores. LOD score was considered “suggestive” if > = 1.6 (p = 0.63) and “significant” if > = 3.55 (p = 0.05). Threshold values for linkage were determined by a 1,000 permutation test by using J/qtl.

### Culture of bone-marrow derived macrophage and RAW264.7 macrophage cell line

To generate bone marrow-derived macrophages (BMDMs), bone marrow progenitor cells were harvested from mice and cultured for 7 days in 70% (vol/vol) D10 (DMEM containing 10% (vol/vol) FBS, 2 mM glutamine, 100 µg/ml streptomycin, and 100 units/ml penicillin) and 30% (vol/vol) L-929 cell culture supernatant. Mature BMDMs were washed twice with cold DPBS, collected with 5 mM EDTA in DPBS, and re-plated on tissue-culture plates as reported before [15]. The murine RAW 264.7 macrophage cells (ATCC) were cultured in D10 in tissue culture plates before downstream experiments.

### Macrophage phagocytosis assay

The day before treatment, 2×10^6^ BMDMs from either C57BL/6J or A/J were seeded to 6-well plate for analysis of *S. aureus* phagocytosis ability. Next day BMDMs were either treated with fresh medium, TNF-α at 100 ng/ml, or *S. aureus* particles at 10 µg/ml for 24 hours. On the day of the phagocytosis experiment, *S. aureus* Sanger 476 were grown to exponential period (OD600≈0.8) and harvested by centrifugation. After washing in DPBS, *S. aureus* were stained by Hexidium Iodide (100 µg/ml) for 15 minutes at room temperature followed by washing once in DPBS and re-suspended in DPBS on ice [59]. Then multiplicity of infection (MOI) 10 was applied for *S. aureus* infection. Briefly, old medium were removed and replaced with fresh medium containing 2×10^7^ *S. aureus* to each well of 6-well plate with BMDMs, followed by quick spin at 500 rpm for 5 minutes at room temperature. Cells were then incubated at 37°C for 30 min in CO_2_ incubator to allow bacterial uptake by macrophages. After introduction of *S. aureus*, trypsin was added at a final concentration of 0.25% for 10 minutes at room temperature to remove any residual bacteria at the macrophage surface. Macrophages were next washed three times with DPBS to remove remaining bacteria and floated by 5 mM EDTA in DPBS. Macrophages were then stained by FITC-F4/80 and analyzed by Flow cytometer [60]. The fluorescence produced from hexidium iodide staining falls into FL2 channel (Excitation 488/Emmision 575) in FACSCanto [59]. Experiments were repeated at least three times.

### Small interfering RNA (siRNA) experiments

To test the role of each candidate gene on cytokine production by host defense cells, we transfected siRNAs into the mouse macrophages. All siRNAs were purchased from Invitrogen. RAW264.7 cells or bone-marrow derived macrophages from C57BL/6J were transfected with 50 nM siRNA by Lipofectamine RNAiMAX (Invitrogen) according to the manufacturer's instructions. Twenty-four hours post-transfection, cells were treated with *S. aureus* Bioparticles (Invitrogen) to a final concentration of 10 µg/ml. At 24 hours post-infection, supernatants were collected and stored at −80°C for Luminex-multiplex cytokine assay. In parallel experiments, cells at 3 hours post-infection were harvested for RNA extraction by RNeasy (QIAGEN), RT-PCR by SuperScript II (Invitrogen) and SYBR Green qPCR analysis (ABI) respectively. Experiments were repeated at least three times. A full list of gene names and siRNA ID numbers were in Table S1.

### Luciferase assay

The day before transfection, 5×10^6^ RAW264.7 macrophages were seeded into 10 cm dishes. On the second day, 10 µg NF-κB-Luc (Clontech) and 0.5 µg pRL-TK (Promega) plasmids were co-transfected by Lipofectamine LTX (Invitrogen) according to manufacturer's instruction. At 6 hours post-transfection, RAW264.7 were split into 6-well plate (1×10^6^/well) and cultured overnight in CO_2_ incubator. On the third day, scrambled siRNA or siRNA for each candidate gene was transfected into 6-well plate by Lipofectamine RNAiMAX according to manufacturer's instruction. At 6 hours post-transfection, cells were split into 24-well plate (4×10^5^/well). On the fourth day, cells were stimulated by either D10 or D10 containing *S. aureus* lipoteichoic acid (LTA) (10 µg/ml) or *S. aureus* particles (10 µg/ml) for seven hours. Then cells were lysed by 1×Passive Lysis Buffer (Promega) and luciferase activities were analyzed by dual-luciferase reporter assay system (Promega). All of Firefly luciferase activity was normalized by the Renilla luciferase activity and relative fold changes were compared. Experiments were repeated at least three times.

### Inhibition of NF-κB signaling

4×10^5^ RAW264.7 macrophages were seeded into each well in 24-well plate and cultured overnight in CO_2_ incubator. Then cells were treated by D10 with DMSO or 4 µM Bay 11-7085 (EMD Millipore) for one hour. Afterwards, the cells were stimulated by four different ways including D10+DMSO, D10+DMSO+*S. aureus* particles (10 µg/ml), D10+2 µM Bay and D10+2 µM Bay+*S. aureus* particles (10 µg/ml) for 3 hours. Then RNA was extracted, and reverse-transcription PCR and qPCR were applied. Experiments were repeated at least three times.

### Measurement of cytokine/chemokine production

Cytokine production was assayed from the collected supernatant of both *S. aureus*-challenged siRNA transfected RAW cells and the *S. aureus*-challenged BMDM from C57BL/6J and CSS11 using multiplex cytokine assay kit (Invitrogen) and Luminex technology available at Duke Human Vaccine Institute.

### Quantitative PCR

Total RNA was isolated using RNeasy kits (Qiagen) primed with random hexamer oligonucleotides and reversely transcribed using Invitrogen SuperScript II. Real-time quantitative PCR was performed using SYBR Green Mastermix (ABI). All data were normalized to 18s rRNA.

### Western blot

Cells were lysed in RIPA buffer with cocktail of proteinase and protein phosphatase inhibitors. Then 20 µg whole cell lysate was loaded to SDS-PAGE and transferred to PVDF membrane. Blotting was followed according to manufacturer's instruction.

### Statistical analyses

The differences in candidate gene expression, mRNA and protein of cytokines and chemokines and luciferase activities were analyzed by two-tailed Student's *t* test. The difference in mice survival rate was analyzed by Mann-Whitney *u* test. P-values smaller than 0.05 were considered to be statistically significant.

## Supporting Information

Figure S1 **A/J and CSS11 are susceptible to** ***E. coli*** **infection as compared with C57BL/6J mice.** C57BL/6J, A/J, or CSS11 mice were injected (i.p.) with *E.coli* (K1H7) at 2×10^5^ CFU/g (n = 10 for each strain). Comparison of survival curves was performed by Mann-Whitney *u* test. The difference between C57BL/6J and CSS11 mice was significant (p<0.05).
(TIF)

Figure S2 **Quantitative PCR of the six candidate genes in either uninfected or** ***E. coli*** **infected A/J and C57BL/6J mice.** Both eight-week-old male A/J and C57BL/6J mice were injected (i.p.) with *E. coli* at 1×10^7^ CFU/g or DPBS (n = 6 each). At two hours post infection blood RNA were extracted by QIAGEN RNeasy Protect Animal Blood Kit, followed by reverse-transcription PCR and SYBR-green quantitative-PCR. The expression of all target genes was normalized to 18s rRNA.
(TIF)

Figure S3 **Knockdown efficiency in RAW264.7 macrophages.** RAW264.7 cells were transfected by either scramble siRNA or siRNA of *Dcaf7*, *Dusp3*, *Fam134c*, *Psme3,* and *Slc4a1*. At 24 hours post-transfection RNA was extracted followed by reverse-transcription PCR, qPCR and normalization to 18s rRNA.
(TIF)

Figure S4 **Knockdown efficiency in bone marrow derived macrophages.** BMDMs from C57BL/6J were transfected by either scramble siRNA or siRNA of *Dusp3* and *Psme3*. At 24 hours post-transfection RNA was extracted followed by reverse-transcription PCR, qPCR and normalization to 18s rRNA.
(TIF)

Figure S5 **The phenotype of bone-marrow derived macrophages from CSS11 and C57BL/6J have no detectable difference.** The bone-marrow derived macrophages from both C57BL/6J or CSS11 mice were stained with FITC-F4/80 and PE-CD11b and analyzed by FACScanto. As shown no detectable difference was observed.
(TIF)

Figure S6 **Luminex-multiplex cytokine assay of bone-marrow derived macrophages from both C57BL/6J and CSS11.**
(TIF)

Figure S7 **Luminex-multiplex cytokine assay of knockdown RAW264.7 macrophages transfected with five candidate genes.** VEGF was dramatically elevated in *Dusp3* and *Psme3* knockdown RAW264.7 cells in both uninfected and *S. aureus* infected conditions.
(TIF)

Figure S8 **Cytokine assay of BMDMs transfected with either scrambled,** ***Dusp3*** **or** ***Psme3*** **siRNA after** ***S. aureus*** **infection.** No detectable difference was observed of the production of GM-CSF and IL-1β in *Dusp3* siRNA or *Psme3* siRNA transfected BMDMs as compared with scrambled siRNA control.
(TIF)

Figure S9 **Quantitative PCR heat map of cytokines and chemokines from RAW264.7 macrophages transfected with candidate gene siRNA and BMDMs from C57BL/6J and CSS11.** (**A**) Higher levels of cytokines and chemokines from BMDMs from CSS11 mice as compared to C57BL/6J mice. (B) Knockdown *Dusp3* and *Psme3* in RAW264.7 macrophages enhanced the expression of most cytokines and chemokines.
(TIF)

Figure S10 **Pre-exposure to** ***S. aureus*** **reduced phagocytosis ability in both C57BL/6J and A/J BMDMs.** Pre-exposure of BMDMs of both C57BL/6J and A/J to TNF-α (100 ng/ml) for 24 hours enhanced the phagocytosis ability of both strains. Pre-exposure of BMDMs to *S. aureus* particles (10 µg/ml) for 24 hours reduced the phagocytosis ability, and the reduction is higher in BMDMs from A/J as compared with C57BL/6J.
(TIF)

Figure S11 **Expression pattern of** ***Dusp3*** **and** ***Psme3*** **in human neutrophils and macrophages stimulated by** ***S. aureus*** **.** Human neutrophil data from public data set GEO:GSE16837 (http://www.ncbi.nlm.nih.gov/geo/query/acc.cgi?acc=GSE16837) was analyzed. *Dusp3* increased to 9.88 fold at 3 hr (p<0.001) and 7.95 fold at 6 hr (p<0.05) as compared with 0 hr after *S. aureus* stimulation. *Psme3* decreased to 0.68 fold at 3 hr (p<0.0001) and 0.45 fold at 6 hr (p<0.0001) as compared with 0 hr. Human macrophage data from public data set GEO:GSE13670 (http://www.ncbi.nlm.nih.gov/geo/query/acc.cgi?acc=GSE13670) was analyzed. *Dusp3* increased to 1.62 fold at 8 hr (p<0.005) compared with controls; and *Psme3* decreased to 0.73 fold at 8 hr (p<0.001) as compared with each control.
(TIF)

Table S1 **List of qPCR primers for candidate genes.**
(TIF)

Table S2 **List of siRNAs used in this study.**
(TIF)

Table S3 **List of antibodies used in this study.**
(TIF)

Table S4 **List of qPCR primers for cytokines and chemokines applied used in this study.**
(TIF)
