# Correction: *Dusp3* and *Psme3* Are Associated with Murine Susceptibility to *Staphylococcus aureus* Infection and Human Sepsis

**DOI:** 10.1371/journal.ppat.1004259

**Published:** 2014-06-25

**Authors:** The PLOS Pathogens Staff

The published article contains three errors that the authors wish to correct.

There is an error in one of the author names. The tenth author's middle initial is omitted. The correct name is: Joshua T. Thaden.

There is a contribution missing from the Acknowledgments. The omitted statement can be seen here:

We thank Dr. Deepak Voora for providing access to the gene expression data from the healthy human subjects used as controls in this study.

A citation and corresponding reference is omitted from a sentence in the “Human subjects” sub-section of the “Methods”. The sentence should read:

Healthy controls were defined as uninfected human (n =  43), enrolled as part of a study on the effect of aspirin on platelet function among healthy volunteers (Voora et al, 2013).

The reference is:

Voora D, Cyr D, Lucas J, Chi JT, Dungan J, et al. (2013) Aspirin exposure reveals novel genes associated with platelet function and cardiovascular events. J Am Coll Cardiol 62: 1267-76. doi: 10.1016/j.jacc.2013.05.073
