# Supplementary figures and images for: Dusp3 and Psme3 Are Associated with Murine Susceptibility to Staphylococcus aureus Infection and Human Sepsis
Source: PLoS Pathog. 2014 Jun 5;10(6):e1004149. doi: 10.1371/journal.ppat.1004149 (PMC4047107; doi:10.1371/journal.ppat.1004149)

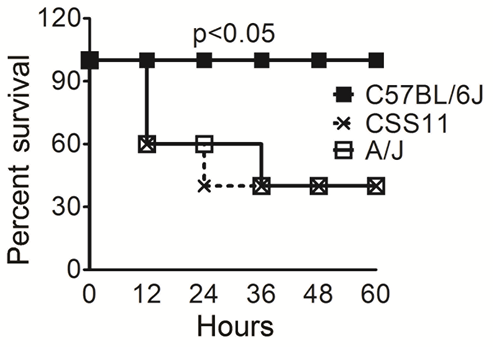

Supplement: Figure S1 — A/J and CSS11 are susceptible to E. coli infection as compared with C57BL/6J mice. C57BL/6J, A/J, or CSS11 mice were injected (i.p.) with E.coli (K1H7) at 2×10^5^ CFU/g (n = 10 for each strain). Comparison of survival curves was performed by Mann-Whitney u test. The difference between C57BL/6J and CSS11 mice was significant (p<0.05). (TIF) [file ppat.1004149.s001.tif]

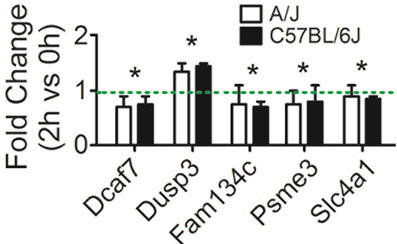

Supplement: Figure S2 — Quantitative PCR of the six candidate genes in either uninfected or E. coli infected A/J and C57BL/6J mice. Both eight-week-old male A/J and C57BL/6J mice were injected (i.p.) with E. coli at 1×10^7^ CFU/g or DPBS (n = 6 each). At two hours post infection blood RNA were extracted by QIAGEN RNeasy Protect Animal Blood Kit, followed by reverse-transcription PCR and SYBR-green quantitative-PCR. The expression of all target genes was normalized to 18s rRNA. (TIF) [file ppat.1004149.s002.tif]

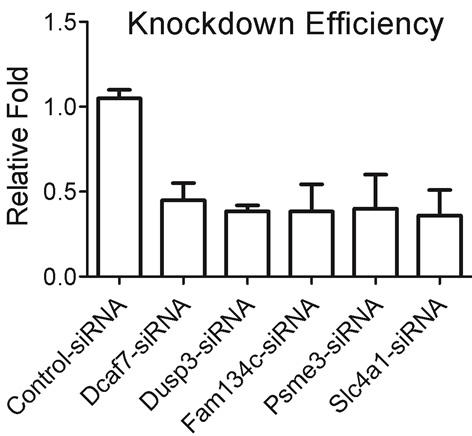

Supplement: Figure S3 — Knockdown efficiency in RAW264.7 macrophages. RAW264.7 cells were transfected by either scramble siRNA or siRNA of Dcaf7, Dusp3, Fam134c, Psme3, and Slc4a1. At 24 hours post-transfection RNA was extracted followed by reverse-transcription PCR, qPCR and normalization to 18s rRNA. (TIF) [file ppat.1004149.s003.tif]

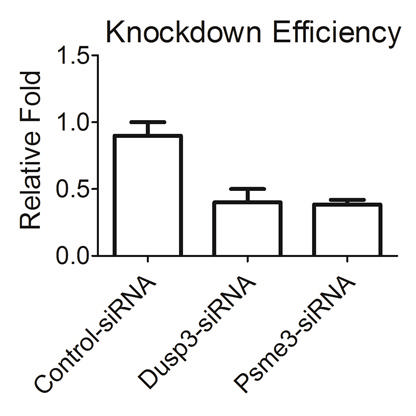

Supplement: Figure S4 — Knockdown efficiency in bone marrow derived macrophages. BMDMs from C57BL/6J were transfected by either scramble siRNA or siRNA of Dusp3 and Psme3. At 24 hours post-transfection RNA was extracted followed by reverse-transcription PCR, qPCR and normalization to 18s rRNA. (TIF) [file ppat.1004149.s004.tif]

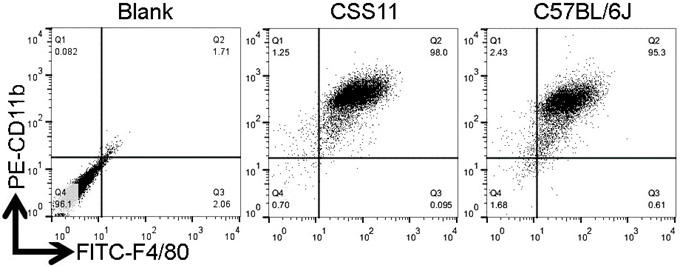

Supplement: Figure S5 — The phenotype of bone-marrow derived macrophages from CSS11 and C57BL/6J have no detectable difference. The bone-marrow derived macrophages from both C57BL/6J or CSS11 mice were stained with FITC-F4/80 and PE-CD11b and analyzed by FACScanto. As shown no detectable difference was observed. (TIF) [file ppat.1004149.s005.tif]

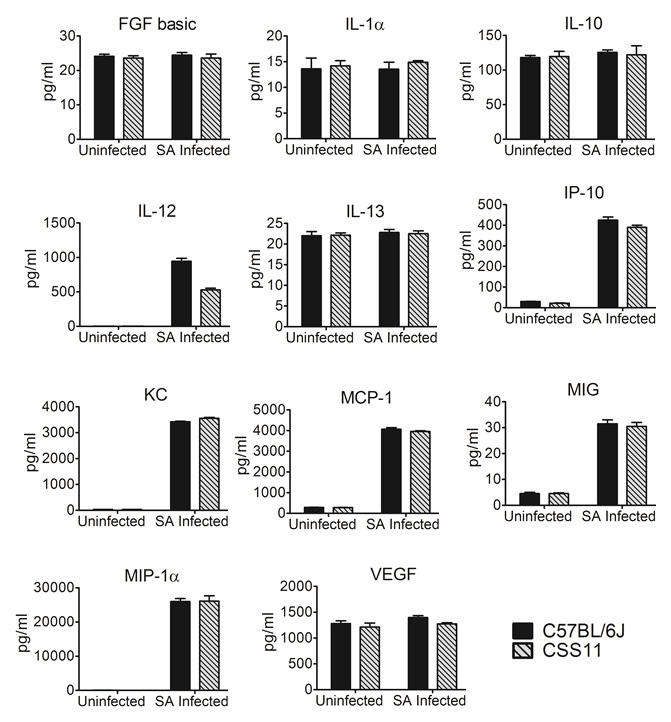

Supplement: Figure S6 — Luminex-multiplex cytokine assay of bone-marrow derived macrophages from both C57BL/6J and CSS11. (TIF) [file ppat.1004149.s006.tif]

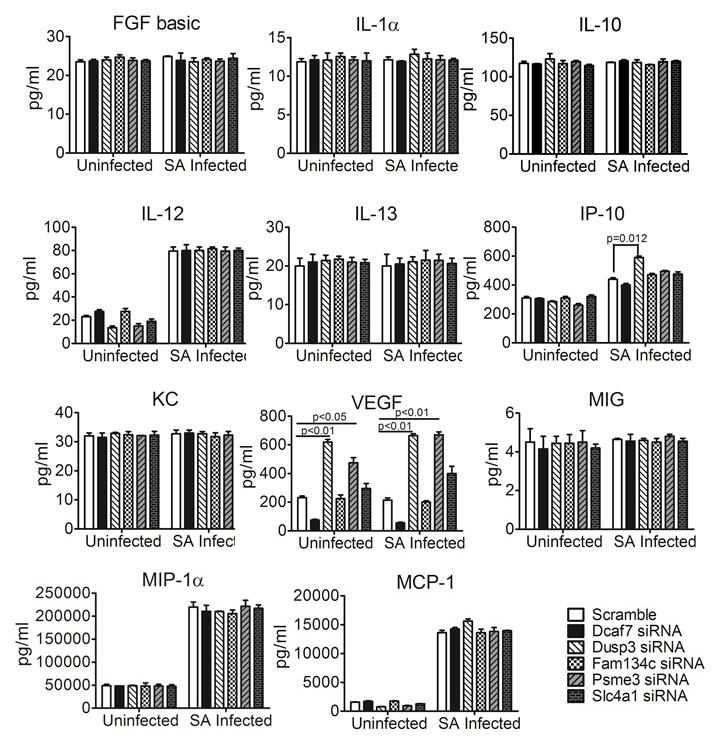

Supplement: Figure S7 — Luminex-multiplex cytokine assay of knockdown RAW264.7 macrophages transfected with five candidate genes. VEGF was dramatically elevated in Dusp3 and Psme3 knockdown RAW264.7 cells in both uninfected and S. aureus infected conditions. (TIF) [file ppat.1004149.s007.tif]

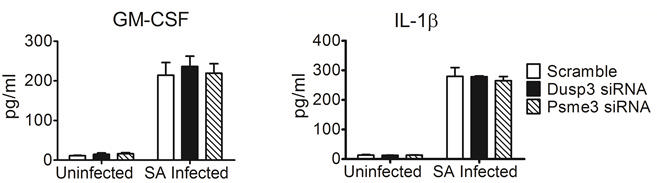

Supplement: Figure S8 — Cytokine assay of BMDMs transfected with either scrambled, Dusp3 or Psme3 siRNA after S. aureus infection. No detectable difference was observed of the production of GM-CSF and IL-1β in Dusp3 siRNA or Psme3 siRNA transfected BMDMs as compared with scrambled siRNA control. (TIF) [file ppat.1004149.s008.tif]

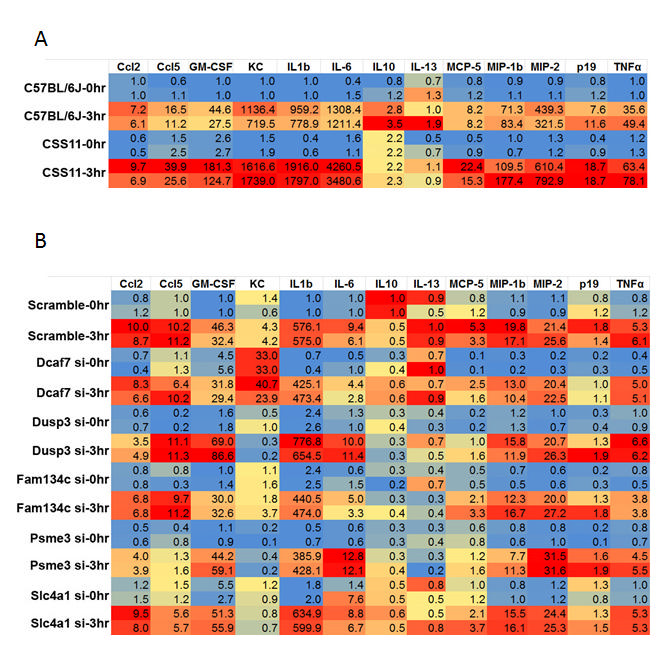

Supplement: Figure S9 — Quantitative PCR heat map of cytokines and chemokines from RAW264.7 macrophages transfected with candidate gene siRNA and BMDMs from C57BL/6J and CSS11. (A) Higher levels of cytokines and chemokines from BMDMs from CSS11 mice as compared to C57BL/6J mice. (B) Knockdown Dusp3 and Psme3 in RAW264.7 macrophages enhanced the expression of most cytokines and chemokines. (TIF) [file ppat.1004149.s009.tif]

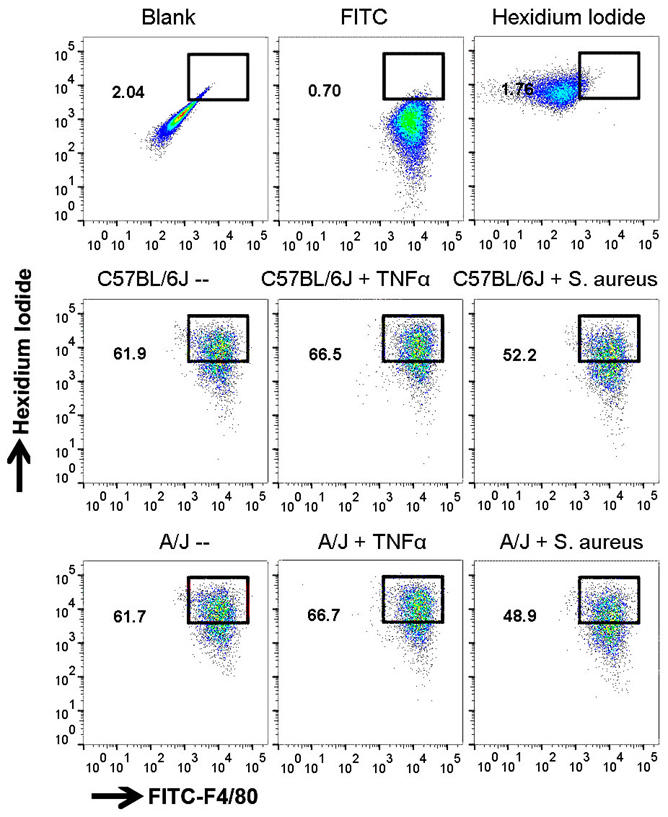

Supplement: Figure S10 — Pre-exposure to S. aureus reduced phagocytosis ability in both C57BL/6J and A/J BMDMs. Pre-exposure of BMDMs of both C57BL/6J and A/J to TNF-α (100 ng/ml) for 24 hours enhanced the phagocytosis ability of both strains. Pre-exposure of BMDMs to S. aureus particles (10 µg/ml) for 24 hours reduced the phagocytosis ability, and the reduction is higher in BMDMs from A/J as compared with C57BL/6J. (TIF) [file ppat.1004149.s010.tif]

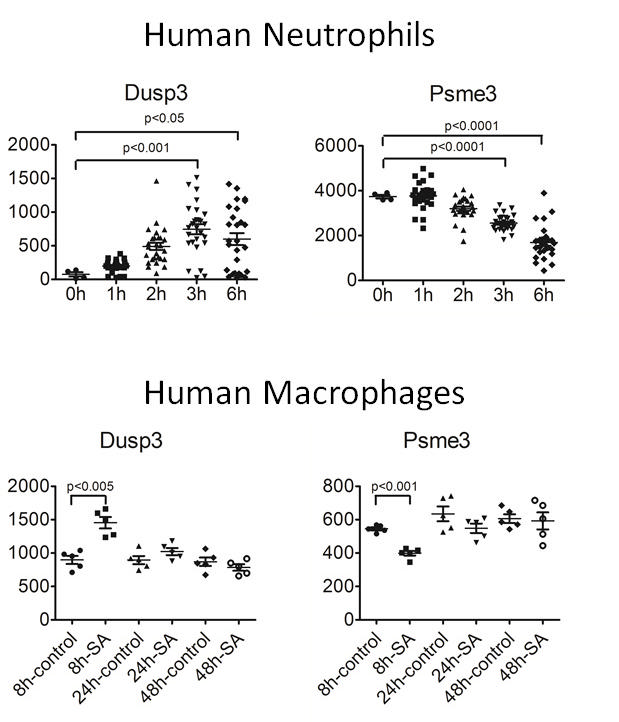

Supplement: Figure S11 — Expression pattern of Dusp3 and Psme3 in human neutrophils and macrophages stimulated by S. aureus . Human neutrophil data from public data set GEO:GSE16837 (http://www.ncbi.nlm.nih.gov/geo/query/acc.cgi?acc=GSE16837) was analyzed. Dusp3 increased to 9.88 fold at 3 hr (p<0.001) and 7.95 fold at 6 hr (p<0.05) as compared with 0 hr after S. aureus stimulation. Psme3 decreased to 0.68 fold at 3 hr (p<0.0001) and 0.45 fold at 6 hr (p<0.0001) as compared with 0 hr. Human macrophage data from public data set GEO:GSE13670 (http://www.ncbi.nlm.nih.gov/geo/query/acc.cgi?acc=GSE13670) was analyzed. Dusp3 increased to 1.62 fold at 8 hr (p<0.005) compared with controls; and Psme3 decreased to 0.73 fold at 8 hr (p<0.001) as compared with each control. (TIF) [file ppat.1004149.s011.tif]

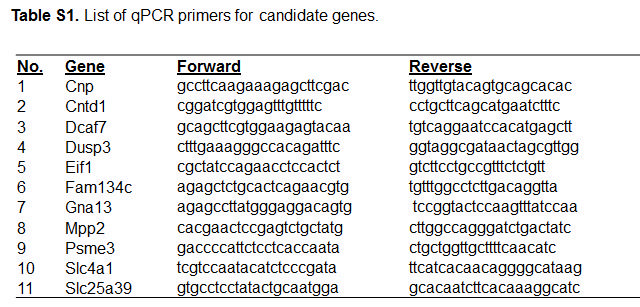

Supplement: Table S1 — List of qPCR primers for candidate genes. (TIF) [file ppat.1004149.s012.tif]

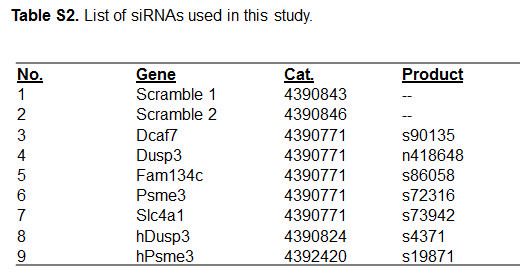

Supplement: Table S2 — List of siRNAs used in this study. (TIF) [file ppat.1004149.s013.tif]

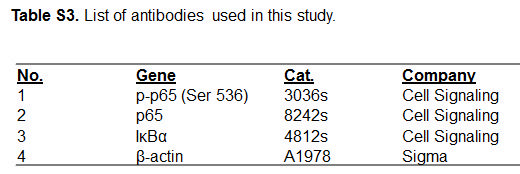

Supplement: Table S3 — List of antibodies used in this study. (TIF) [file ppat.1004149.s014.tif]

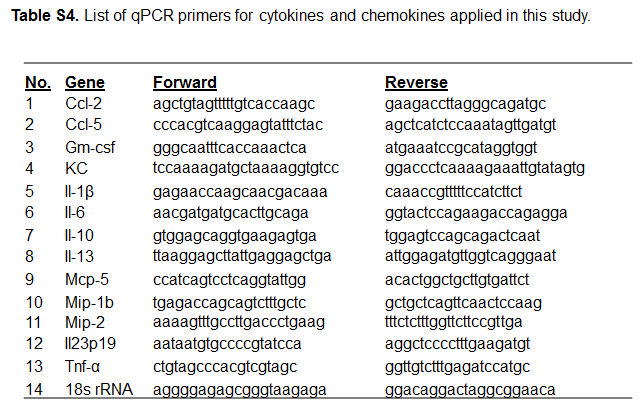

Supplement: Table S4 — List of qPCR primers for cytokines and chemokines applied used in this study. (TIF) [file ppat.1004149.s015.tif]
